# Evaluating a condition-specific PROM for amelogenesis imperfecta in children and young people: insights from patients and clinicians

**DOI:** 10.1007/s40368-026-01214-x

**Published:** 2026-05-11

**Authors:** J. Altaher, S. Parekh, F. Ryan

**Affiliations:** University College London (UCL), London, UK

**Keywords:** Amelogenesis imperfecta, PROMs, Paediatric dentistry, Quality of Life, Enamel defects

## Abstract

**Purpose:**

Amelogenesis imperfecta (AI) is a genetic enamel defect affecting both primary and permanent dentitions, often leading to functional, aesthetic, and psychosocial challenges. Patient-reported outcome measures (PROMs) are increasingly used in paediatric dentistry. This study aimed to explore the impact of AI on oral health-related quality of life in children and adolescents using an AI-specific PROM, and to assess clinician use of the PROM in the UK.

**Methods:**

This single-centre retrospective service evaluation involved children with AI who completed a nine-question PROM pre- and mid-treatment. Responses were analysed by treatment stage, age group, and AI subtype. Clinician feedback on the AI PROM was obtained via an online survey.

**Results:**

A total of 68 paired AI PROMs from patients aged 7–19 years were analysed at pre-treatment and mid-treatment stages. The cohort comprised 40 females (58.8%) and 28 males (41.2%). Children under 13 years were more frequently reported functional difficulties, such as pain and eating challenges, while adolescents (≥ 13 years) more often expressed psychosocial concerns including bullying and low self-confidence more frequently. Satisfaction with dental appearance increased from 26 to 42% by the mid-treatment stage. In the clinician feedback survey, 23 of 78 paediatric dentists responded (29.5% response rate). Of those familiar with the AI PROM, 67% reported actively using it in their clinical practice.

**Conclusion:**

The AI PROM, while non-validated, provided meaningful insights into patient experiences and supported more empathetic, patient-centred care. Incorporating clinician perspectives further supported its perceived relevance and usefulness.

## Introduction

Amelogenesis imperfecta (AI) is a hereditary enamel defect that can present as missing, thin, or discoloured enamel due to abnormalities in the quantity and/or quality of enamel formation. In addition to clinical challenges, AI can significantly affect a child’s oral function, appearance, and self-esteem, all of which contribute to a reduced oral health-related quality of life (OHRQoL) (Parekh et al. 2014; Genderson et al. 2013; Jokovic et al. 2002; Crawford et al. 2007). Children; ; ; and adolescents with AI may experience sensitivity, pain, aesthetic concerns, and bullying (Broutin et al. 2023), particularly during school years.

AI is genetically heterogeneous, with pathogenic variants identified in genes involved in enamel matrix secretion, mineralisation, and maturation, including AMELX, ENAM, MMP20, KLK4, and FAM83H among others (Bloch-Zupan et al. 2023; Smith et al. 2017). Disruptions in these pathways result in either quantitative enamel defects (hypoplastic forms) or qualitative mineralisation defects (hypomature and hypocalcified phenotypes), which differ in clinical presentation, symptom burden, and treatment complexity. As AI requires staged, often lifelong management, its impact extends beyond isolated clinical episodes.

To capture these subjective impacts, a condition-specific PROM for AI was developed, piloted, and implemented across four UK specialist paediatric centres (Lyne et al. 2021). Overall, 60 children aged 5–17 years completed the PROM with 72% reporting pain or sensitivity, and 76% expressed dissatisfaction with the appearance of their teeth. Notably, satisfaction improved with treatment stage—81% of mid-treatment patients reported being happy with their teeth, compared to 33% at pre-treatment.

While validated instruments such as the Child Oral Health Impact Profile (COHIP) and ECOHIS are widely used to assess paediatric OHRQoL, these generic tools are not designed to capture the enamel-specific challenges and prolonged treatment pathways characteristic of AI. Children with AI often experience recurrent restorative interventions, rapid post-eruptive enamel breakdown, aesthetic concerns related to opacity or discolouration, and functional limitations linked to mineralisation defects. A condition-specific PROM may therefore provide greater granularity in identifying AI-related functional and psychosocial impacts and better support personalised care planning across a longitudinal treatment journey.

Although the AI PROM has not yet undergone formal psychometric validation, it has demonstrated clinical utility in surfacing patient-specific concerns and guiding treatment discussions. This study builds on that work by evaluating the AI-specific PROM at a single UK paediatric centre, analysing responses from children and young persons at two stages of treatment. Additionally, it incorporates clinician feedback to assess the tool’s perceived utility and limitations in everyday clinical practice.

## Methods

This retrospective service evaluation was conducted at a UK specialist paediatric dental centre and was registered with the local clinical governance department. As a service evaluation using routinely collected data, formal ethical approval was not required. Records from children and young persons (CYP) with a confirmed diagnosis of amelogenesis imperfecta (AI) who attended specialist clinics between January 2022 and January 2024 were included. PROMs were completed as part of routine care and retrieved from electronic health records. Inclusion criteria required both a pre-treatment and a mid-treatment PROM to be fully completed and available in the records. Given the chronic and lifelong nature of AI management, these two stages were defined pragmatically. For clarity, “pre-treatment” referred to the stage prior to commencement of restorative or aesthetic interventions beyond preventive measures. “Mid-treatment” included patients who had commenced or completed anterior restorative phases (e.g. resin composite buildups, whitening) and/or were undergoing staged posterior management, including preventive or temporisation phases, but had not reached a definitive endpoint of care. The term *mid-treatment* was adopted rather than *post-treatment* because AI care typically involves staged interventions and ongoing review, making it difficult to define a single endpoint. In this context, *mid-treatment* encompassed patients who were actively receiving care as well as those who had completed their main restorative phase and were under recall. This approach therefore reflects a service-based snapshot of progress within an ongoing continuum of care, rather than a definitive completion point.

The initial PROM pilot study (Lyne et al. 2021) was conducted across four UK specialist centres, including Eastman Dental Hospital (EDH). In contrast, the current study is limited to patients seen at EDH only. The same electronic health record system was used, and some overlap with previously studied patients is possible. However, the authors did not have access to the raw data from the initial study, so exact overlap could not be confirmed. Importantly, the current cohort primarily comprises new patients followed over a longer period, offering novel longitudinal insights.

Data were extracted and recorded in Microsoft Excel™ (manufacturer’s details), including demographic details, AI subtype, and treatment history.

Amelogenesis imperfecta cases were categorised into hypoplastic, hypocalcified, and hypomature subtypes, following established clinical diagnostic criteria based on enamel appearance and texture. Hypoplastic AI was characterised by reduced enamel thickness with a hard, smooth surface; hypocalcified AI exhibited normal enamel thickness with soft, chalky, or friable enamel prone to post-eruptive breakdown; and hypomature AI presented with normal enamel thickness but mottled, opaque, or cream-brown enamel that was slightly softer in texture.

Terminology note: In line with recent frameworks that group qualitative enamel defects under the broader term hypomineralised (Bloch-Zupan et al. 2023; Smith et al. 2017), we report the two operational subtypes used in our dataset, hypomature and hypocalcified, as mineralisation-related phenotypes within this category, whilst retaining Witkop terminology in tables and figures for continuity with existing datasets and comparability with prior publications.

A small number of patients presented with mixed clinical features (e.g. hypomature and hypocalcified). Because this subgroup was too small for meaningful statistical analysis (*n* = 4), these cases were excluded from subtype-specific comparisons. Consequently, percentage calculations for AI subtypes were based on the remaining 64 patients who exhibited a single predominant phenotype. Mixed cases were, however, documented descriptively within the demographic overview to maintain transparency of cohort composition.

Descriptive statistics were used to summarise PROM responses, and subgroup analyses explored patterns by age (< 13 vs. ≥ 13 years), AI phenotype, and treatment stage.

The AI PROM consists of nine items with structured response options (“Never”, “Sometimes”, “Often”), allowing ordinal comparison across treatment stages (Readable,. 2020). The questionnaire was self-completed by children and young people during routine clinical visits. For younger participants or those requiring clarification, a parent or clinician could provide neutral assistance limited to reading or explaining the wording without prompting specific responses. Questionnaires were completed prior to treatment discussions and subsequently uploaded to the electronic health record. No defined recall period (e.g. previous four weeks) was specified at the time of administration, reflecting its original service-based design; this has been acknowledged as a limitation and will be addressed in future validation work.

To explore clinician perspectives on the AI PROM, a cross-sectional online survey was developed using Qualtrics™. Ethical approval for the survey was obtained via University College London’s Low-Risk Research Ethics Committee (ID: 26593/001). The 25-item survey included a mix of closed-ended (multiple choice and Likert scale) and open-ended questions and was structured around key themes: familiarity with the PROM, perceived content relevance, clinical utility, implementation challenges, and recommendations for improvement. Survey questions were informed by a literature review and refined in collaboration with the research team. The final version was piloted with paediatric dental staff and reviewed for readability (MacDonald et al. 2012). The survey was distributed via email to 78 dentists through the Amelogenesis Imperfecta Clinical Excellence Network (AICEN). Use of this targeted mailing list ensured that respondents were clinicians who routinely manage children and young people with AI, thereby providing relevant and experienced feedback. Participation was voluntary, and confidentiality was maintained; no IP addresses or personally identifiable data were collected via the Qualtrics™ platform. A Participant Information Leaflet and an electronic consent form were embedded at the start of the survey. Quantitative data were analysed descriptively within Qualtrics™ and Excel™, while qualitative responses were examined thematically using a content analysis approach.

## Results

### Participant characteristics

A total of 68 children and young people (CYP) with paired pre- and mid-treatment AI PROM responses were included in the analysis. The age range was 7–19 years. The mean age at first PROM completion was 12 years, and the mean age at most recent PROM was 13 years, with an average interval of approximately 10 months between assessments. The cohort comprised 40 females (58.8%) and 28 males (41.2%). AI phenotypes included hypomature (*n* = 49; 72%), hypoplastic (*n* = 9; 13%), hypocalcified (*n* = 6; 9%), and mixed phenotypes (*n* = 4; 6%). Mixed phenotypes were excluded from subtype-specific percentage analyses, as described in the Methods section.

### Patient-reported outcomes

**Fig. 1:**
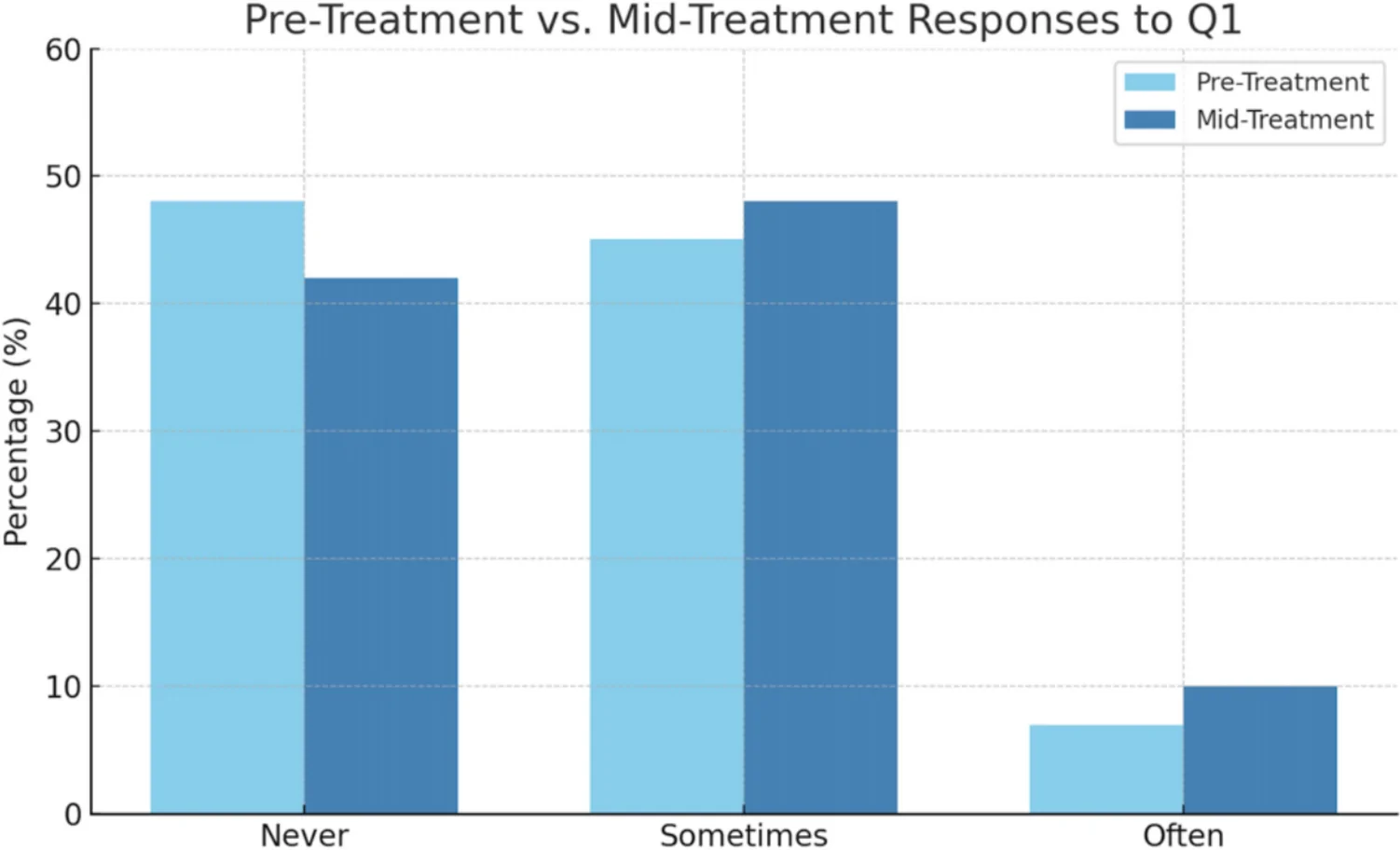
Responses to PROM Question 6: “Do your teeth affect your confidence to smile?” comparing pre-treatment and mid-treatment results

**Fig. 2:**
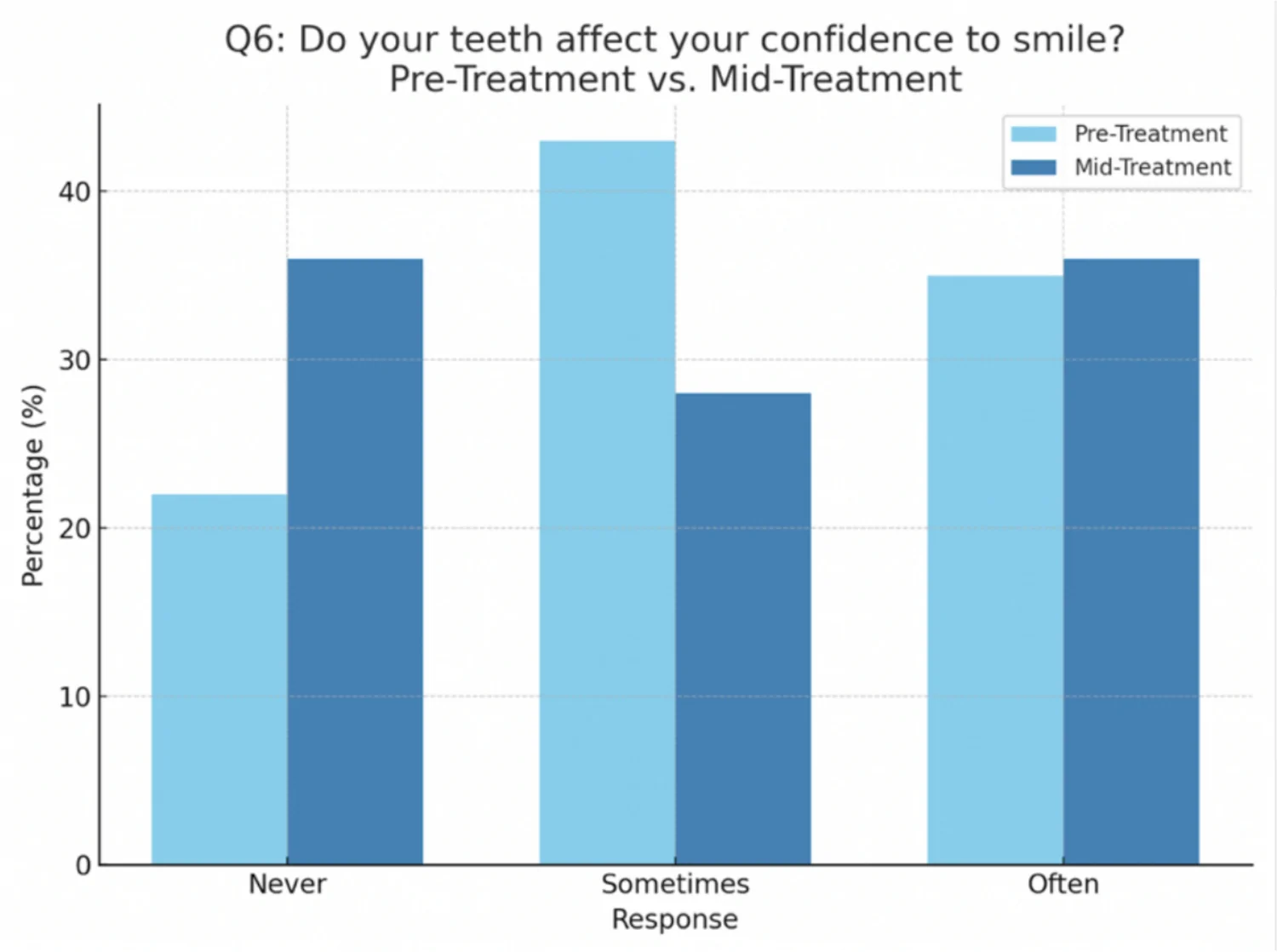
Responses to PROM Question 1: “Do your teeth cause you pain or sensitivity?” pre-versus mid-treatment

**Fig. 3:**
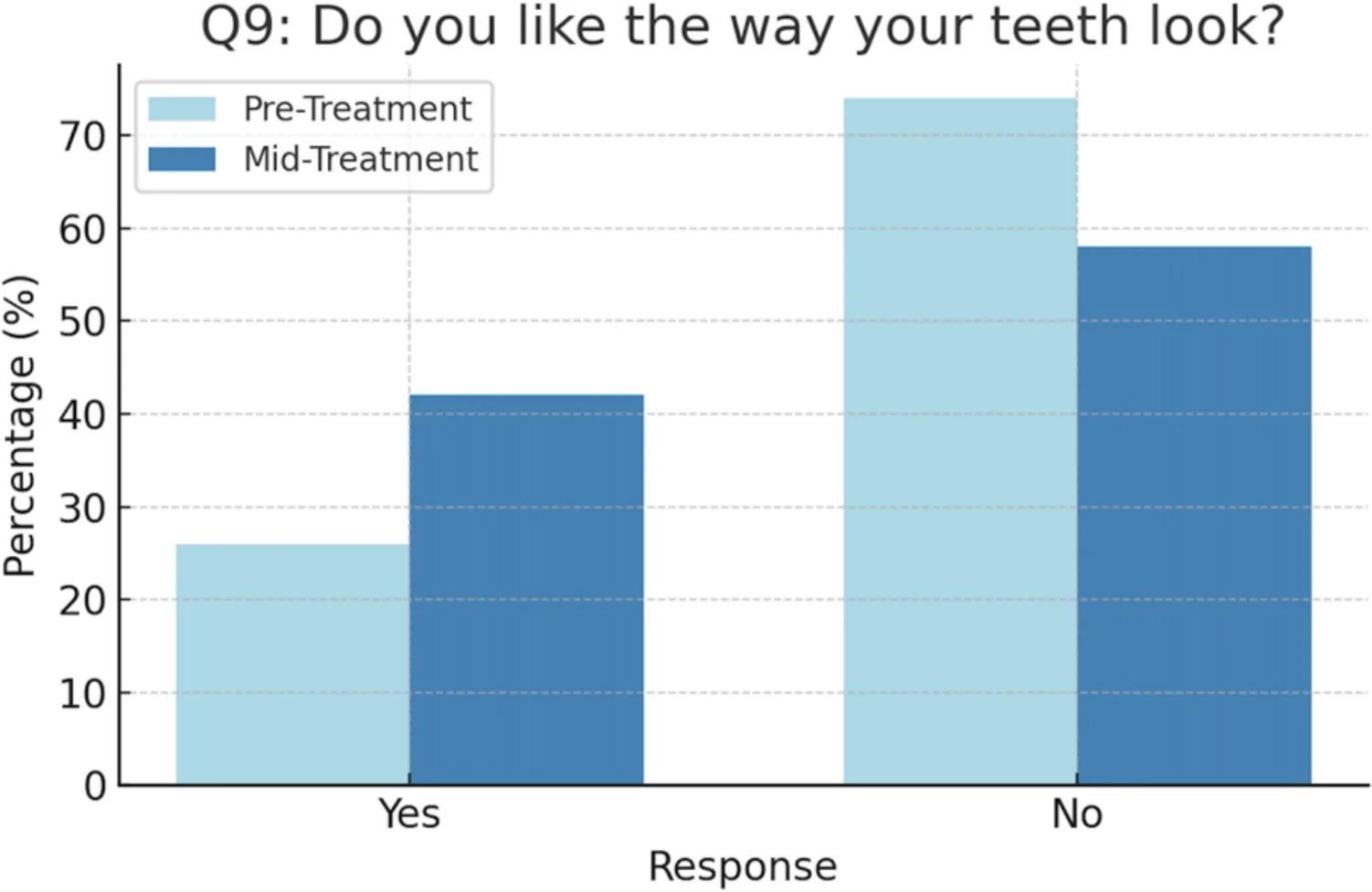
Responses to PROM Question 9: “Do you like the way your teeth look?” pre- versus mid-treatment

At pre-treatment, 78% of children reported that their teeth affected their confidence to smile (i.e. responded ‘Sometimes’ or ‘Often’), compared to 64% at mid-treatment (Fig. 1). Pain and sensitivity responses showed mixed results: although some children reported improved comfort, the proportion reporting frequent pain increased slightly from 7 to 10% (Fig. 2). Aesthetic dissatisfaction was particularly common among adolescents. Satisfaction with dental appearance improved from 26% at pre-treatment to 42% at mid-treatment (Fig. 3). Brushing discomfort and school absences remained largely unchanged. Younger children (< 13 years) showed more noticeable improvements in functional areas such as eating and hygiene.

### Subgroup trends

**Fig. 4:**
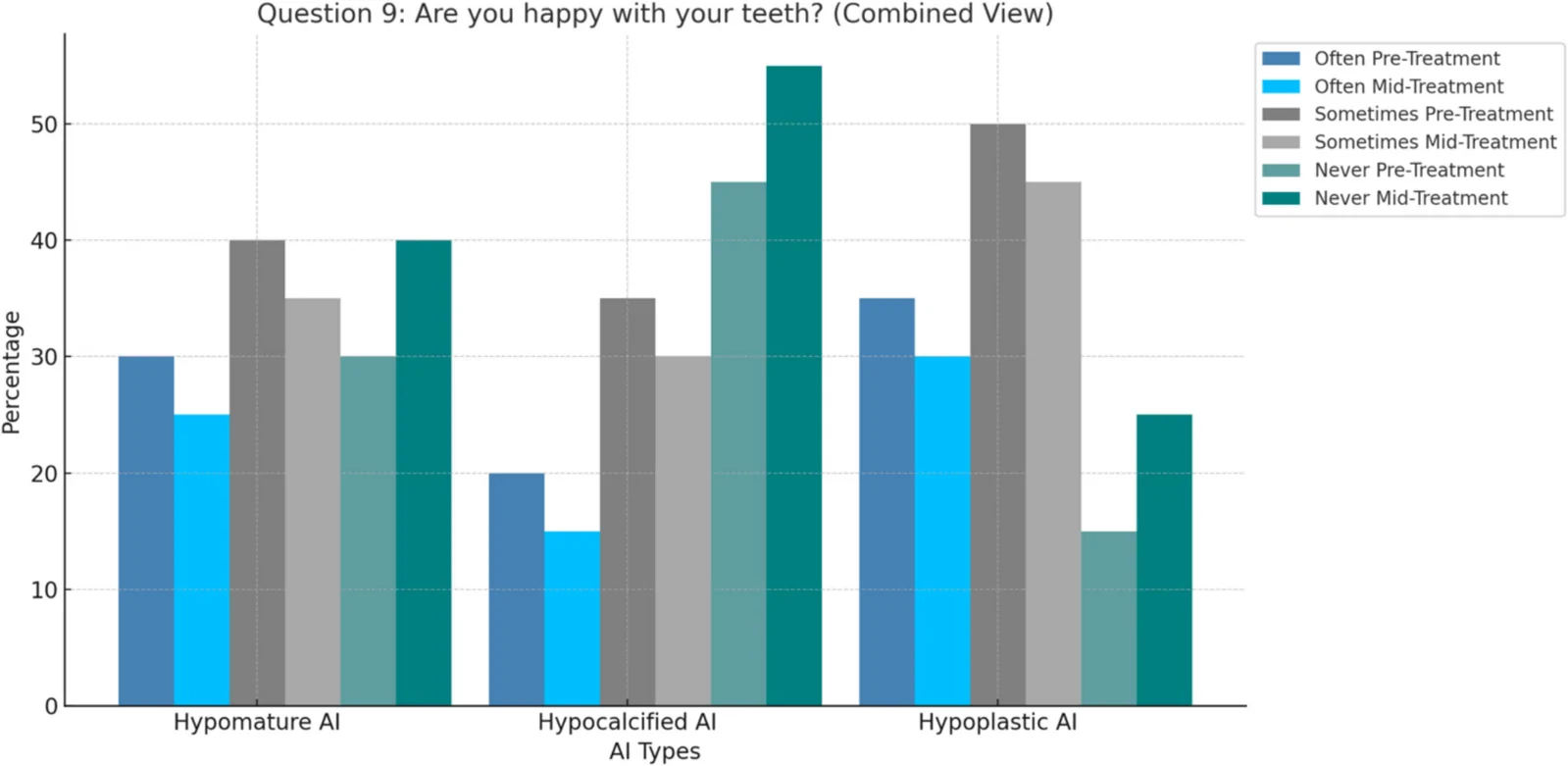
Patient responses to PROM Question 9 (“Are you happy with your teeth?”) stratified by AI phenotype (Hypomature, Hypocalcified, Hypoplastic) and treatment stage. Responses are shown across three categories (Often, Sometimes, Never) for both pre-treatment and mid-treatment stages

**Fig. 5:**
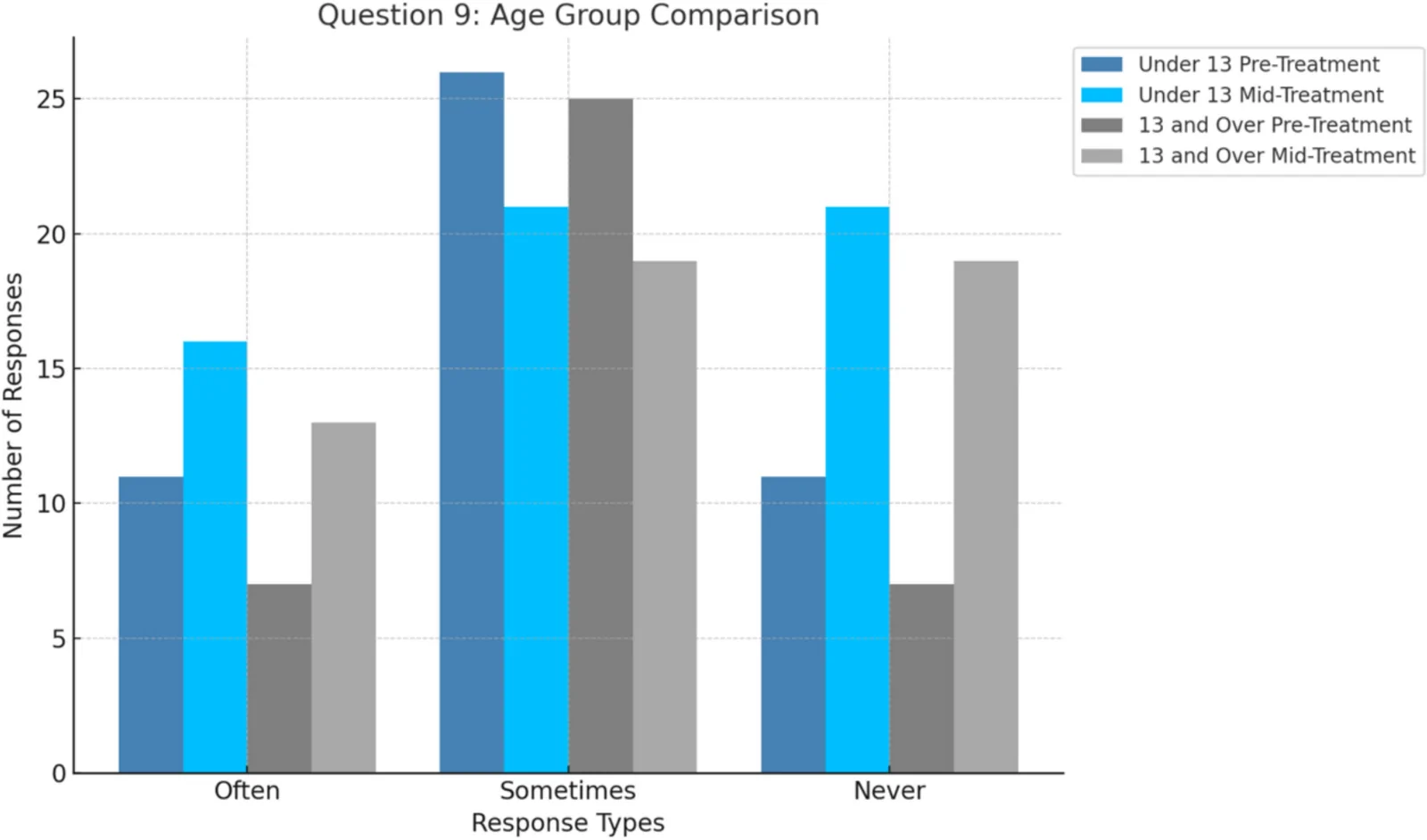
Patient responses to PROM Question 9 (“Are you happy with your teeth?”) grouped by age (< 13 vs. 13 and over) and treatment stage. The figure highlights differences in perception of satisfaction between age groups at pre- and mid-treatment time points

Subgroup analysis by AI phenotype and age suggested general trends, although numbers were too small to do formal statistical analysis. Of the 68 participants, four (5.9%) exhibited mixed phenotypic characteristics and were therefore excluded from subtype-specific comparisons. Percentages presented below are based on the remaining 64 patients with a single predominant phenotype. Children with hypomature AI (72%) made up the largest subgroup and showed some improvements in satisfaction and confidence mid-treatment (Fig. 4). Those with hypocalcified AI (9%) experienced persistent sensitivity, while those with hypoplastic AI (13%) continued to report low confidence despite the majority of anterior restorations having been done in that specific subgroup. Younger children (< 13 years) tended to report greater functional improvements (Fig. 5), whereas adolescents remained more affected by psychosocial factors.

### Treatment modalities and patient perception

Common treatments included tooth whitening (26%), fissure sealants (18%), and anterior resin composites (11%). Whitening was associated with increased confidence, particularly in adolescents with hypomature AI. Preventive measures such as fluoride varnish and desensitising toothpastes were used across all subtypes. Full-coverage restorations, including stainless steel crowns, were used in only four patients. Due to the small sample size, these cases were not included in the main treatment analysis to avoid overinterpretation or statistical bias. The focus remained on more commonly delivered treatments to ensure findings were representative of the broader cohort. Children who received a combination of aesthetic and functional treatments generally reported greater satisfaction at mid-treatment.

### Clinician use and perceptions of the AI PROM (Qualtrics™ survey)

A total of 23 paediatric clinicians consented to participate in the online survey. Of these, 82% (18/22) reported familiarity with the AI PROM, and among them, 67% (12/18) confirmed using it in their clinical unit. All respondents supported its use at the pre-treatment and review stages, while 67% also endorsed its use mid-treatment, particularly in longer or multi-phase care plans.

Clinicians highlighted several benefits of the PROM. 64% reported that it improved communication with patients and families, and 56% felt it enhanced patient satisfaction with their treatment experience. Notably, 83% said it improved their understanding of the emotional and functional impact of AI on children’s lives. However, the average usefulness rating was modest, 56 out of 100, suggesting room for refinement.

### Clinician recommendations for improvement

Clinicians provided extensive qualitative feedback suggesting improvements to enhance the AI PROM’s clinical relevance and usability. Four key themes emerged from the content qualitative analysis (Table 1). Many respondents (44%) advocated expanding the PROM to capture broader treatment outcomes, including emotional and long-term effects. Others recommended streamlining question design, allowing mid-treatment assessments, and improving digital accessibility through mobile platforms or integration with electronic health records (Table 2).

**Table 1:** Full list of AI PROM questions

Question no	Question text:
1	Do your teeth cause you pain or sensitivity?
2	Do you have difficulty eating certain foods because of your teeth?
3	Do you feel different from your friends because of your teeth?
4	Have you ever been bullied or teased because of your teeth?
5	Do you avoid going to the dentist because of how your teeth are?
6	Do your teeth affect your confidence to smile?
7	Do you avoid brushing your teeth because it hurts or feels uncomfortable?
8	Have your teeth ever caused you to miss school?
9	Do you like the way your teeth look?

**Table 2:** Clinician recommendations to improve the AI PROM

Recommendation theme	Clinicians mentioning (%)	Example quote
Expand treatment outcomes	44% (*n* = 11)	“Include questions about emotional impacts and long-term QoL”
Streamline question content design	36% (*n* = 9)	“Focus on severity rather than frequency and reduce the number of questions”
Flexible timing for assessments	36% (*n* = 9)	“Introduce mid-treatment assessments to monitor progress and adapt treatment plans”
Improved accessibility	28% (*n* = 7)	“A mobile app or integration into EHRs would make it easier to use in a busy clinic”

## Discussion

The AI PROM, though non-validated, provided valuable insights into the lived experiences of children with AI, revealing improvements in satisfaction and confidence during treatment—particularly among younger patients and those with hypomature AI. However, challenges such as persistent sensitivity, aesthetic dissatisfaction, and psychosocial impacts remained, especially in older children and those with hypocalcified AI. This could be because treatment for most patients was still on-going. Furthermore, the results support the growing view that condition-specific PROMs add granularity to routine clinical review by surfacing psychosocial concerns (confidence, bullying, treatment anxiety) (Coffield et al. 2005; Hashem et al. 2013; Broutin et al. 2023) that may not track linearly with clinical milestones. This echoes earlier work showing that children with AI experience substantial OHRQoL burdens across function and appearance domains, and that perceived benefit often emerges in phases as restorative and preventive care proceed.

Because AI requires lifelong, phased interventions, a clear endpoint for post-treatment evaluation is difficult to define. As such, the ‘mid-treatment’ time point was used to reflect progress at the time of data collection, even if patients had transitioned to review. This pragmatic approach aligns with the chronic and evolving nature of AI care. Our “mid-treatment” framing is intentionally service-based: it captures early patient-perceived change during active care or early recall, rather than implying a definitive outcome. This choice aligns with the literature on lifelong AI management, in which interventions are staged and periodically revisited as dentitions develop (Patel et al. 2013; MacDonald et al. 2012).

Post-treatment PROM data were not collected, as children with AI often face a prolonged and complex treatment journey involving lifelong follow-up, staged interventions, and substantial care burdens (Patel et al. 2013; MacDonald et al. 2012). The mid-treatment time point was therefore chosen to reflect early outcomes within the context of this ongoing, resource-intensive pathway. Future prospective work should evaluate responsiveness (change sensitivity) of the AI PROM across multiple time points (e.g. pre-treatment, early review, post-phase completion) and explore minimal important differences for key items (confidence to smile, sensitivity, bullying). Establishing measurement properties (internal consistency, test-retest reliability, construct validity, and responsiveness) will determine the PROM’s suitability for individual care and service evaluation.

Clinician feedback reinforced the PROM’s clinical relevance, especially in opening conversations around appearance, bullying, and treatment anxieties. Most respondents valued its role in communication and care planning, though barriers such as time constraints, survey length, and limited digital integration were cited. Suggestions for improvement included streamlining the tool, expanding outcome domains, and embedding the PROM into digital platforms to enhance accessibility. These implementation themes mirror broader PROM adoption challenges in paediatric dentistry: tools must be brief, digitally accessible, and clearly interpretable in the chairside workflow (Gilchrist et al. 2020). Our findings suggest three pragmatic enhancements: (1) a short-form version prioritising the highest-yield items (confidence, sensitivity/pain, eating, bullying); (2) optional item branching to maintain brevity when problems are absent; and (3) EHR integration with simple graphical trajectories to support shared decision-making with children and parents.

Although the PROM has not yet undergone formal psychometric validation, this service evaluation incorporated informal assessments through clinician feedback. Their responses provided early insights into face and content validity, indicating perceived clarity, relevance, and value in clinical settings. In parallel, our subgroup patterns are clinically coherent: hypomature AI, typically with normal thickness but qualitative enamel change, showed greater gains in satisfaction mid-treatment (likely reflecting earlier aesthetic wins), whilst hypocalcified AI, soft, friable enamel with post-eruptive breakdown, retained higher sensitivity burdens. Such phenotype-linked differences reinforce the need for phenotype-specific care plans and for PROM reporting that distinguishes functional from psychosocial domains (Bloch-Zupan et al. 2023; Smith et al. 2017).

AI is classically divided into hypoplastic, hypomature, and hypocalcified categories; however, mixed forms are recognised in clinical practice. In the present cohort, mixed phenotypes were identified but were too few (*n* = 4) for meaningful subgroup analysis; they were therefore excluded from subtype-specific percentages (denominator = 64) whilst documenting them descriptively. It is acknowledged that recent literature, including Bloch-Zupan et al. (2023), increasingly uses the term hypomineralised as an umbrella for qualitative enamel defects (Bloch-Zupan et al. 2023; Smith et al. 2017). Within this broader category, our hypomature and hypocalcified subtypes represent distinct mineralisation-stage phenotypes, reflecting differences in enamel crystal growth and maturation, whereas hypoplastic represents a primarily quantitative thickness defect. To maintain consistency with prior datasets and tables, we have retained the original Witkop terminology but note this conceptual overlap and plan to harmonise future reporting accordingly.

This dual perspective, combining patient and clinician insights, strengthens the case for condition-specific PROMs in paediatric dental care. While broadly validated tools like COHIP and ECOHIS remain valuable (Broder et al. 2012), the AI PROM captures the unique burden and treatment outcomes associated with AI specifically. Its tailored focus highlights its promise as a developmental yet meaningful adjunct to routine care (Patel et al. 2013; MacDonald et al. 2012; Gilchrist et al. 2020). Looking ahead, successful integration will rely on digital delivery, child-friendly design, psychometric testing, and continued refinement to ensure these tools are both practical and impactful in everyday practice. Importantly, plans are underway to administer the AI PROM as part of a prospective, multi-centre study in collaboration with the AI Clinical Excellence Network and academic partners. This will enable formal psychometric testing, including internal consistency, test-retest reliability, construct validity, and responsiveness to change. A prospective, multi-centre study with harmonised terminology and a pre-registered validation plan is now warranted to confirm measurement properties and clinical utility.

## Limitations

This evaluation has several limitations. The small sample sizes, both in patient and clinician datasets, limit generalisability and preclude formal statistical comparisons, particularly within AI subgroups. As a retrospective study relying on self-reported data, responses may have been influenced by recall bias, mood, or parental assistance. The mid-treatment PROM time point was chosen due to the ongoing nature of AI care, which typically involves lifelong follow-up and staged interventions, making a definitive “post-treatment” stage difficult to define. The AI PROM was introduced recently, restricting longitudinal data collection and follow-up. Additionally, the clinician survey had a low response rate (29.5%), was UK-focused and excluded patient/parent perspectives, limiting its scope. Future studies should prioritise prospective, multi-centre designs and include diverse views to enhance robustness and relevance. In addition, as questionnaires were completed within the clinical setting and occasional parental or clinician clarification may have occurred, responses may have been influenced by social desirability bias.

## Conclusion

Combining both patient and clinician perspectives, this evaluation highlights the emerging role of a condition-specific, non-validated AI PROM in paediatric dentistry. PROM responses revealed meaningful improvements in satisfaction and confidence, particularly in younger patients and those with hypomature AI, while also surfacing ongoing challenges like sensitivity and social anxiety—especially in hypocalcified cases. Clinicians valued the PROM’s ability to open conversations around hidden concerns, such as bullying and treatment-related anxiety (Coffield et al. 2005; Hashem et al. 2013; Broutin et al. 2023), but cited implementation barriers including time constraints and the need for digital integration. This dual insight reinforces the PROM’s potential as a patient-centred communication tool, offering tailored information beyond routine clinical assessments. While broader validation is needed, the AI PROM complements established OHRQoL measures making it a promising adjunct for condition-specific care (Broder et al. 2012; Gilchrist et al. 2020). A planned multi-centre study will aim to formally validate the tool and optimise its integration into practice.

## Data Availability

No datasets were generated or analysed during the current study.
